# Investigation of the Shear Behavior of Concrete Beams Reinforced with FRP Rebars and Stirrups Using ANN Hybridized with Genetic Algorithm

**DOI:** 10.3390/polym15132857

**Published:** 2023-06-28

**Authors:** Bo Di, Renyuan Qin, Yu Zheng, Jiamei Lv

**Affiliations:** 1 School of Environment and Civil Engineering, Dongguan University of Technology, Dongguan 523808, China; dibo@dgut.edu.cn (B.D.); qinry@dgut.edu.cn (R.Q.); 18022490249@163.com (J.L.); 2 Guangdong Provincial Key Laboratory of Intelligent Disaster Prevention and Emergency Technologies for Urban Lifeline Engineering, Dongguan 523808, China

**Keywords:** FRP, shear capacity, coupling effect, neural network, genetic algorithm

## Abstract

The shear strength prediction of concrete beams reinforced with FRP rebars and stirrups is one of the most complicated issues in structural engineering applications. Numerous experimental and theoretical studies have been conducted to establish a relationship between the shear capacity and the design variables. However, existing semi-empirical models fail to deliver precise predictions due to the intricate nature of shear mechanisms. To provide a more accurate and reliable model, machine learning (ML) techniques are adopted to study the shear behavior of concrete beams reinforced with FRP rebars and stirrups. A database consisting of 120 tested specimens is compiled from the reported literature. An artificial neural network (ANN) and a combination of ANN with a genetic optimization algorithm (GA-ANN) are implemented for the development of an ML model. Through neural interpretation diagrams (NID), the critical design factors, i.e., beam width and effective depth, shear span-to-depth ratio, compressive strength of concrete, FRP longitudinal reinforcement ratio, FRP shear reinforcement ratio, and elastic modulus of FRP longitudinal reinforcement rebars and FRP stirrups, are identified and determined as input parameters of the models. The accuracy of the proposed models has been verified by comparing the model predictions with the available test results. The application of the GA-ANN model provides better statistical results (mean value *V*_exp_/*V*_pre_ equal to 0.99, R^2^ of 0.91, and RMSE of 22.6 kN) and outperforms CSA S806-12 predictions by improving the R^2^ value by 18.2% and the RMSE value by 52.5%. Furthermore, special attention is paid to the coupling effects of design parameters on shear capacity, which has not been reasonably considered in the models in the literature and available design guidelines. Finally, an ML-regression equation considering the coupling effects is developed based on the data-driven regression analysis method. The analytical results revealed that the prediction agrees with the test results with reasonable accuracy, and the model can be effectively applied in the prediction of shear capacity of concrete beams reinforced with FRP bars and stirrups.

## 1. Introduction

Fiber-reinforced polymer (FRP) composites have been widely applied in the construction industry to repair/strengthen concrete structures, due to their high strength-to-weight ratio, good fatigue properties, environmental insensitivity, etc. [1,2,3]. Recently, FRP has been considered as an alternative material to steel reinforcements in the form of FRP rebars and stirrups in new construction projects in aggressive environments, such as off-shore structures [4], bridge decks [5], and roadbeds [6], to address the corrosion issues of steel reinforcements. However, due to the low elastic modulus, anisotropy, and fracture brittleness of FRP materials, one that should be considered is the shear behavior of FRP-reinforced concrete (FRP-RC) members, which shows significant variation from conventional steel reinforced concrete (RC) members [7,8,9]. In the case of FRP-RC beams, the deformation and crack width are larger than that of the steel RC beams at the same applying loads. Consequently, the shear resistance provided by both uncracked concrete in the compression zone and aggregated interlock is smaller, which results in a lower shear resistance contribution provided by concrete [10,11]. Additionally, bending FRP rebars to form stirrups significantly reduces the strength at the bend zones due to their anisotropic properties, thus resulting in a lower stirrup shear resistance contribution [12,13]. Hence, the overall shear resistance of concrete beams reinforced with FRP rebars and stirrups varies significantly from that of concrete beams reinforced with steel reinforcement. Therefore, it is crucial to accurately predict the shear capacity of FRP-RC beams to avoid catastrophic shear failure that occurs without any prior signs of damage.

Over the last two decades, a considerable number of experimental investigations have been conducted to study the shear behavior of concrete beams reinforced with FRP bars and stirrups [14,15,16,17]. A variety of design parameters that may affect the shear behavior and ultimate strength of FRP-RC beams were considered, such as beam length (L), beam width ($b_{w}$), effective depth (d), shear span-to-depth ratio (a/d), compressive strength of concrete ($f_{c}^{\prime }$), FRP longitudinal reinforcement ratio ($\rho_{fl}$), FRP shear reinforcement ratio ($\rho_{fv}$), elastic modulus of FRP longitudinal reinforcement rebars ($E_{fl}$) and FRP stirrups ($E_{fv}$), ultimate tensile strength of FRP longitudinal reinforcement rebars ($f_{ful}$) and the straight portion of FRP stirrups ($f_{fuv}$), and FRP reinforcing materials (AFRP, BFRP, CFRP, and GFRP). This encouraged the development of semi-empirical models for predicting the shear capacity of FRP-RC beams. The shear strength prediction of concrete beams reinforced with FRP rebars and stirrups is known as one of the most complicated issues in structural engineering applications, and most existing analytical models take account of the ultimate shear capacity of the FRP-RC beam as the linear combination of shear contributions provided by concrete and FRP stirrups, which follows the same design principle as conventional steel reinforced concrete structures. Anisotropy and fracture brittleness of FRP materials and coupling effects among the design parameters in FRPRC structures have not been addressed, resulting in less prediction accuracy using analytical methods [18,19,20,21]. Generally, these prediction models were derived through regression analysis based on limited available test data; therefore, their applicability for a large range of tests is questionable. Razaqpur and Spadea [22] compared the actual shear capacity of 86 FRP-RC beams with their predicted values based on the current design equations provided by different design standards and guidelines. It was found that the mean of the ratio of the test to predicted shear capacity ranges from 0.78 to 2.67, indicating that some of the existing design equations are over-conservative, whereas others sometimes yield un-conservative results. It is necessary to improve understanding of the interaction among various design parameters in relation to the shear behavior and ultimate strength of FRP-RC beams.

With the development of comprehensive experimental data, machine learning (ML)-based techniques are being applied in the field of civil engineering, particularly for FRP-RC structures [23,24,25]. Numerous ML algorithms have been utilized to investigate the mechanical and structural behavior of FRP-RC structures, especially for issues with complexity and fluctuation nature, such as FRP–concrete interfacial bond strength evaluation [26,27], shear strength estimation of FRP in strengthened RC beams [28,29], and shear capacity prediction of FRP-RC beams [30,31]. For example, Jahangir and Eidgahee [26] proposed a bond strength model between FRP composites and the concrete substrates by developing an optimized artificial neural network (ANN) using an artificial bee colony algorithm. Naderpour and Alavi [28] provided a fuzzy-based model for predicting the shear contribution of FRP in RC beams strengthened by externally bonded FRP sheets. Furthermore, several studies have been performed to investigate the shear behavior of FRP-RC beams using ML-based techniques [32,33,34,35,36,37]. In 2011, Kara [32] utilized gene expression programming (GEP) to obtain a shear capacity prediction model for FRP-RC beams without stirrups, where it was shown that the GEP model performed better as compared to the available shear design guidelines. Furthermore, Lee and Lee [33], Jumaa and Yousif [34], and Naderpour et al. [35] developed ANN models to evaluate the shear capacity of FRP-RC beams without stirrups. Moreover, Golafshani and Ashour [36] proposed a new model using biogeography-based programming (BBP) to predict the shear capacity of FRP-RC beams without stirrups based on an experimental database of 138 test specimens. In 2022, Wakjira et al. [37] explored the application of ML in predicting the shear capacity of FRP-RC beams. They focused on the comparison of the performance of different ML techniques including support vector machine (SVM), decision tree (DT), random forest (RF), and extreme gradient boosting (xgBoost). However, few results have been reported in the shear behavior of concrete beams reinforced with FRP stirrups. As the key transverse reinforcement in FRPRC beams, the strength of FRP stirrups would vary at different load levels, due to the curling and stretching of the fibers at the bend zone of FRP stirrups, so the interactions between the strength of FRP stirrups and other design parameters become more significant, which has not been addressed in the literature. Owing to the great reliability and promising results of ML techniques in analyzing FRP-RC structures, it has great potential to be further applied in predicting the shear capacity of FRP-RC beams with stirrups by addressing the complex interaction among various design parameters and potential different shear failure mechanism due to the brittleness of FRP stirrups.

In this paper, both ML-based and regression-based models were proposed for predicting the shear capacity of concrete beams reinforced with FRP rebars and stirrups. For this purpose, an updated experimental database of 120 test specimens with 13 variables was firstly compiled from the literature. The critical parameters governing shear capacity of FRP-RC beams were identified using neural interpretation diagrams (NID), which are further determined as input parameters of the ML-based models. Both ANN and the combination of ANN with the genetic optimization algorithm (GA-ANN) were utilized for the model development. The accuracy, feasibility, and validity of the proposed model were demonstrated through a comparison with the predictions from established models found in existing design codes and studies. Additionally, a comprehensive parametric study was conducted using the proposed GA-ANN model, and the coupling effect between parameters on shear capacity of FRP-RC beams was further revealed. Finally, a practical shear capacity calculation formula was developed for the design purpose based on data-driven regression analysis (DDRA). Compared with existing design codes, the prediction accuracy on shear capacity of FRP-RC beams is highly improved using the proposed GA-ANN model, through considering the anisotropy and fracture brittleness of FRP materials and coupling effects among the design parameters in FRP-RC beams.

## 2. Overview of Current Shear Design Provisions

With the development and application of FRP reinforcements in concrete structures, several prediction models of the structural performance of FRP-RC structures have been included in various design guidelines. In this study, we considered the design formula for shear capacity of FRP-RC members recommended by the American Concrete Institute ACI 440.1R-15 [18], Canadian Standards Association CSA-S806-12 [19], British Institution of Structural Engineers guidelines BISE-99 [20], and the Japan Society of Civil Engineers JSCE-97 [21] summarized in Table 1, together with several other prediction models proposed by El-Sayed et al. [38], Tottori and Wakui [39], Wegian and Abdalla [40], Nehdi et al. [41], and Deitz et al. [42].

All the available design formulas follow the traditional $V_{c}+V_{s}$ philosophy, while the manner in which the concrete shear contribution $V_{c}$ and the stirrup shear contribution $V_{s}$ are calculated differs significantly. Moreover, most of the design formulas are derived essentially from that of conventional steel RC beams with the consideration of different material properties between FRP and steel reinforcements. These modifications mainly account for the relatively low elastic modulus of FRP bars and the reduction in tensile strength at the bending zones of FRP stirrups. To address the low elastic modulus of FRP bars, the BISE-99 [20], JSCE-97 [21], Tottori and Wakui [39], Wegian and Abdalla [40], and Deitz et al. [42] incorporate an elastic modulus ratio ($E_{fl}/E_{s}$) into their models, where $E_{fl}$ and $E_{s}$ are the elastic modulus of FRP bars and steel reinforcement, respectively. However, the elastic modulus ratio ($E_{fl}/E_{s}$) is raised to different magnitudes in these equations. Furthermore, the modification proposed by the ACI 440.1R-15 [18] and CSA S806-12 [19] only includes the FRP reinforcement axial stiffness ($E_{fl}A_{fl}$), where $A_{fl}$ is the area of longitudinal FRP bars. In order to address the reduced tensile strength at the bending zones of FRP stirrups, both the maximum stress and strain at failure are limited to a lower value, while a significant difference exists among the maximum values recommended by different design models.

## 3. Methodology

### 3.1. Experimental Database

**Figure 1:**
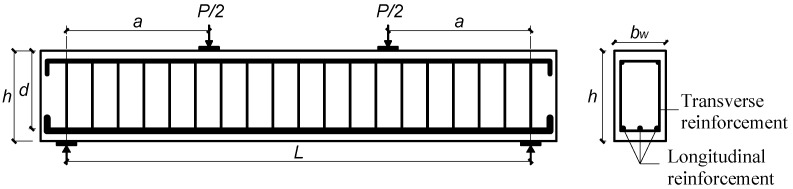
Schematic diagram of concrete beam reinforced with FRP rebars and stirrups.

A database that contains comprehensive and accurate experimental test results is required for developing an efficient and reliable ML-based model. In this regard, an updated experimental database including over 150 data was collected from the literature for the first time [14,15,16,17,22,39,43,44,45,46,47,48,49,50,51,52,53,54,55,56,57,58,59]. The specimens with shear span-to-depth ratios between 1.0 and 4.5 were selected, while deep and slender beams were omitted from the database due to the different design scenarios and failure modes. Eventually, a refined database consisting of 120 groups of samples was utilized for the development of an ML-based model. All selected specimens are simply supported in either three-point or four-point loading arrangement, reinforced with both FRP longitudinal and transverse shear reinforcement, as depicted schematically in Figure 1.

**Figure 2:**
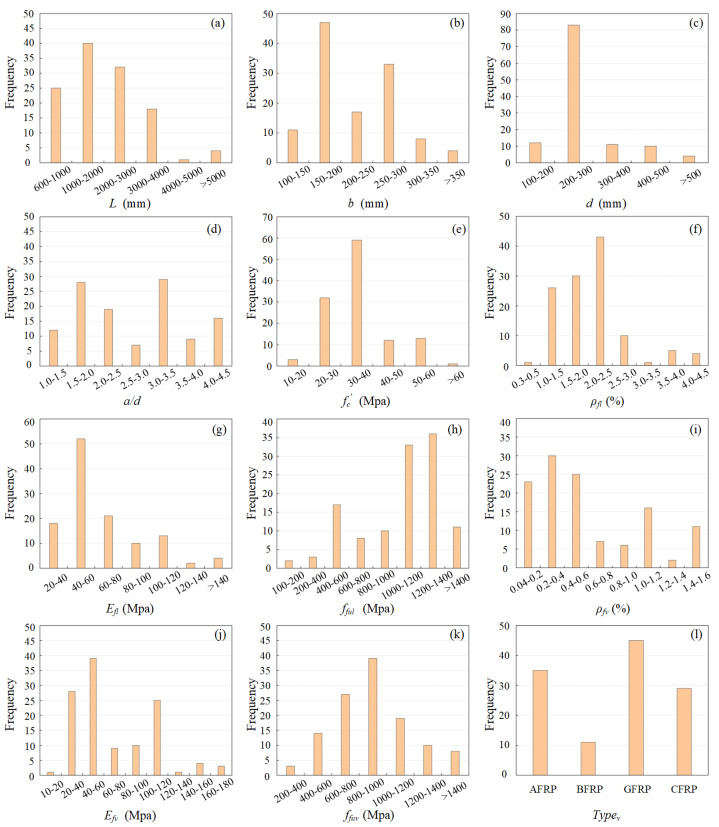
Frequency distribution of the variables in the experimental database.

In total, 13 design factors of FRP-RC beam were investigated in the literature, namely, beam length (L), beam width ($b_{w}$), effective depth (d), shear span-to-depth ratio (a/d), concrete compressive strength ($f_{c}^{\prime }$), FRP longitudinal reinforcement ratio ($\rho_{fl}$), FRP shear reinforcement ratio ($\rho_{fv}$), elastic modulus of FRP longitudinal reinforcement bars ($E_{fl}$) and FRP stirrups ($E_{fv}$), ultimate tensile strength of FRP longitudinal reinforcement bars ($f_{ful}$) and the straight portion of FRP stirrups ($f_{fuv}$), and type of FRP longitudinal reinforcement bars ($Type_{l}$) and FRP stirrups ($Type_{v}$). It should be noted that the contributions of individual parameters to the mechanical performance of FRP-RC beams could be considered variously in different studies, which requires further investigation of the criticality of individual design parameters and their interaction with each other. The details of the experimental data, together with their statistical properties, are presented in Table 2. In addition, the frequency distribution of selected variables in the experimental database is shown in Figure 2, where frequency indicates the number of times that each specific value of the variables appeared in the database. It can be seen that the collected experimental data cover a wide range of design parameters, which can be regarded as proper inputs for the shear capacity model development.

### 3.2. Neural Networks and Genetic Algorithm–Optimized Neural Network

Neural networks (NNs) are information-processing paradigms inspired by biological neural systems, which offer synaptic activity through a matrix of weight updated by the human-like learning process [61]. Artificial neurons are the basic units of NNs, and their typical output can be written as

(1) $$y_{i}=f\left(net_{i}\right)$$

(2) $$net_{i}=\sum_{j=1}^{n}w_{ij}x_{i}-\theta$$

where $x_{i}$ is the input signal from the neuron i, $w_{ij}$ is the connection weight from neuron *j* to neuron i, θ is the threshold, f is the activation function, $y_{i}$ is the output of neuron i.

**Figure 3:**
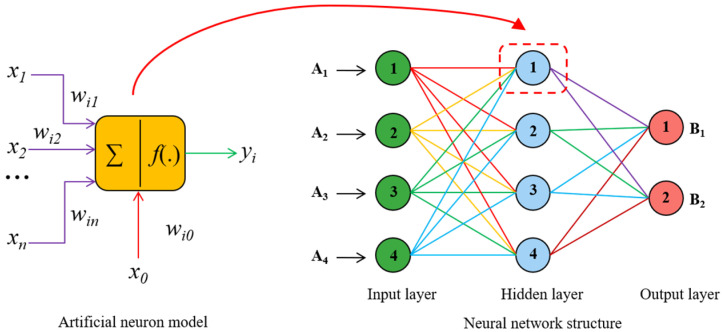
Schematic diagram of artificial neural and NN structure.

A schematic diagram showing the interconnection between the neurons and the typical structure of an NN is illustrated in Figure 3. The input layer, in which the number of neurons is equal to the number of pondered variables, and an output layer are always considered in typical ANN models with a defined number of hidden layers in between. With the increase in hidden layers, the performance of ANN is commonly improved. In this paper, the back-propagation neural network (BPNN) was adopted, whose applicability has been validated in previous studies for the prediction of structural performance of RC structural members. In order to evaluate the accuracy and computational efficiency of an ANN in shear capacity prediction of FRP-RC beams, both single-hidden layer NN and double-hidden layer NN were constructed for the evaluation of their prediction accuracy.

**Figure 4:**
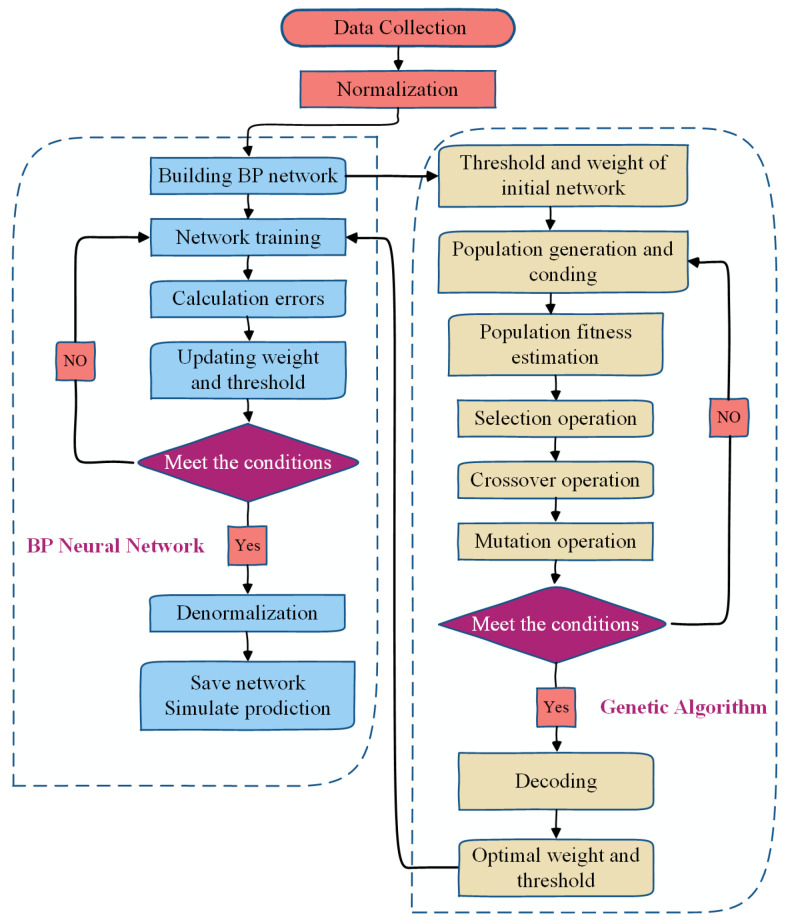
Flowchart of the genetic algorithm-optimized neural network.

In order to further balance the prediction accuracy and efficiency, the initial weights and biases of the ANN should be further optimized for better accuracy and convergency, and genetic algorithms (GAs) are regarded as a potential solution for this purpose [62]. The GA optimizes the parameters and forms codes to interconnect the groups, which distinguishes individuals through operations such as select, cross, and mutation. It uses different adaptation conditions to re-differentiate and screen individuals. In order to optimize the performance of ANN models, a stochastic search algorithm such as GA can be employed to modify the biases and weights of the ANN, which helps to avoid the insufficiency of traditional neural network random assignment and repeated training. Moreover, such a hybrid GA-ANN model has been successfully applied in the prediction and design of engineering structures [63,64,65]. The basic steps of the traditional ANN algorithm and the GA-optimized ANN algorithm are shown in Figure 4.

### 3.3. Parameter Selection and Determination

**Figure 5:**
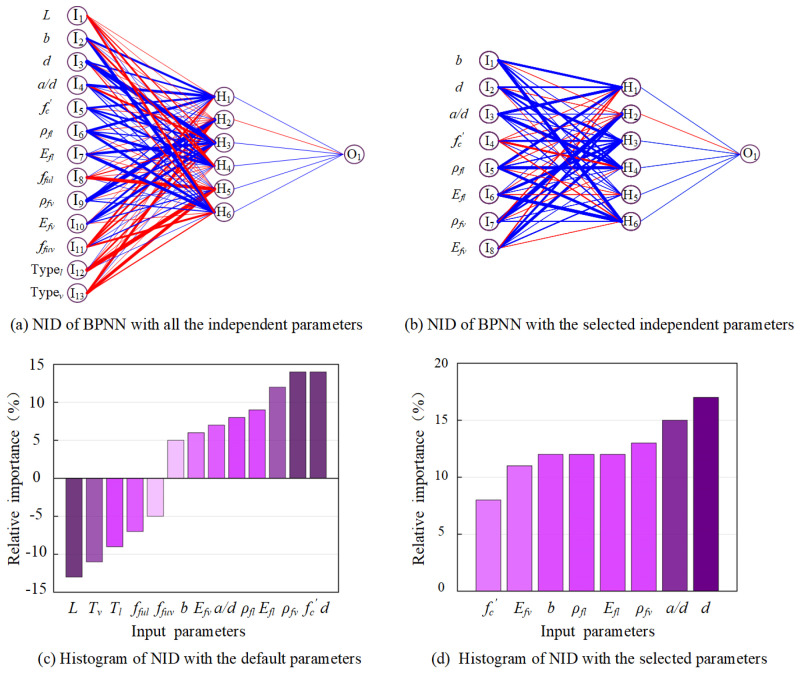
NID and old–style histogram for ANN with all the independent parameters.

The neural interpretation diagram (NID) is proposed to intuitively explain the connection weights between neurons, which helps to clarify the criticality of input parameters on the output of the developed model. In a complete neural network structure, the thickness of each layer of neuron connection lines represents the relative size of each connection weight. As shown in Figure 5a,b, the blue and red lines represent the positive (high influence) and negative (low influence) associations between input and output variables, respectively. By judging the direction (positive and negative) of the two connecting weights from input layer to hidden layer to determine the suppression (different signal) or enhancement (same signal) of the single layer [66,67]. In this study, NID was adopted to confirm the input variables among the 13 parameters from 120 sets of experimental results, namely, beam length (L), beam width (b), effective depth (d), shear span-to-depth ratio (a/d), concrete compressive strength ($f_{c}^{\prime }$), FRP longitudinal reinforcement ratio ($\rho_{fl}$), FRP shear reinforcement ratio ($\rho_{fv}$), elastic modulus of FRP longitudinal reinforcement bars ($E_{fl}$) and FRP stirrups ($E_{fv}$), ultimate tensile strength of FRP longitudinal reinforcement bars ($f_{ful}$) and the straight portion of FRP stirrups ($f_{fuv}$), and type of FRP longitudinal reinforcement bars ($Type_{l}$) and FRP stirrups ($Type_{v}$), to determine the critical parameters that affect the shear capacity of FRP-RC beams. According to the weight matrix between input layer to hidden layer and hidden layer to output layer, the beam width ($b_{w}$), effective depth (d), shear span-to-depth ratio (a/d), concrete compressive strength ($f_{c}^{\prime }$), FRP longitudinal reinforcement ratio ($\rho_{fl}$), FRP shear reinforcement ratio ($\rho_{fv}$), elastic modulus of FRP longitudinal reinforcement bars ($E_{fl}$) and FRP stirrups ($E_{fv}$) are determined as the critical parameters for the proposed ANN model. Furthermore, it is worth noticing that the 8 selected parameters by NID are in accordance with the design parameters suggested in various models, which further demonstrate the correlation between the selected parameters and shear capacity of FRP-RC beams. The relative importance of each variable is obtained based on the algorithm, and its contribution to the output result is determined by judging the relative importance of each parameter and the magnitude of the value. The old-style histogram shown in Figure 5c,d is generated to represent the total weights as a function of relative importance, in which Figure 5c is used to determine the variable with negative importance, and Figure 5d presents the importance of each parameter in terms of percentage.

### 3.4. ANN and GA-ANN Models

**Figure 6:**
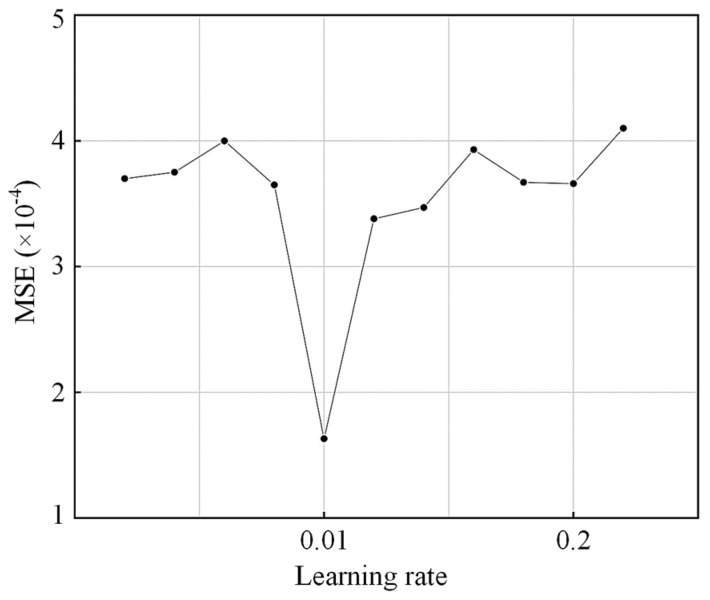
Learning rate parameter determination.

**Figure 7:**
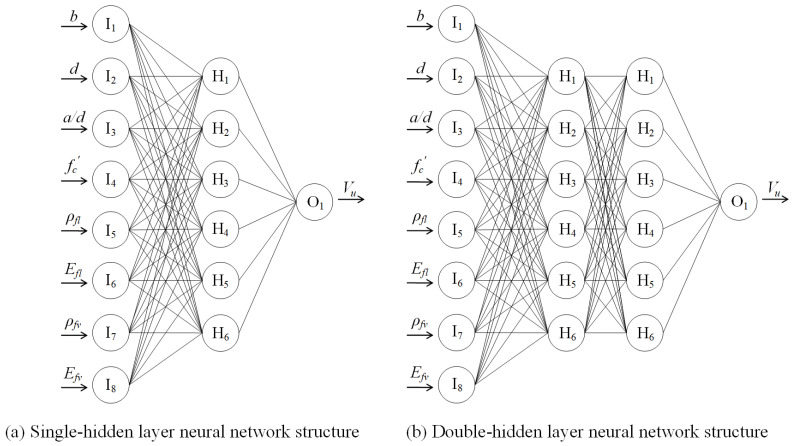
Schematic diagram of the proposed neural network model.

The single-hidden layer, double-hidden layer, and genetic algorithm-optimized neural network models were constructed to predict the shear capacity of concrete beams reinforced with FRP bars and stirrups in this study. After normalizing whole datasets, 86 and 34 sets of test results were randomly selected to train and test the proposed models. The range of the number of hidden layer neurons is judged preliminarily based on Equation (3), and a suitable number of neurons is determined based on the multi-time network training. After several attempts, the number of hidden layer neurons is set as 6 with the learning rate being determined, which is shown in Figure 6. To improve computational efficiency and convergence, the data units were normalized before the network training using Equation (4), and the activation function was selected as sigmoid (x), which is shown in Equation (5), with trainlm being adopted as the learning function. The structure of the neural network model with single-hidden layer, double-hidden layer, and optimization based on genetic algorithm were determined as (8-6-1), (8-6-6-1), and (8-6-1), respectively. A schematic diagram of the proposed neural network model is shown in Figure 7.

(3) $$h=\sqrt{m+n}+a$$

where *h* is the number of hidden layer neurons, *m* is the number of input variables, *n* is the number of output layer nodes, *a* is the adjustment number between 1 and 10.

(4) $$y=0.99\frac{x-x_{\min}}{x_{\max}-x_{\min}}+0.01$$

where *x* is the input value, *x*_min_ is the minimum value of the input number, *x*_max_ is the maximum value of the input number.

(5) $$\mathrm{sigmoid}\left(x\right)=\frac{1}{1+e^{\left(-x\right)}}$$

## 4. Results and Discussion

### 4.1. Prediction of Shear Capacity

**Figure 8:**
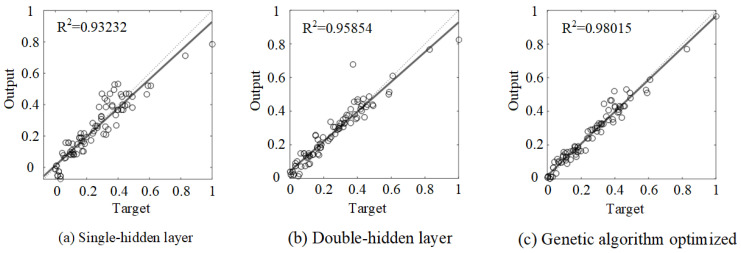
Training results of each neural network model.

The prediction results from the training models are presented in Figure 8. According to the training results of the above models, the overall determination coefficients (R^2^) of the three models for single-hidden layer, double-hidden layer, and genetic algorithm-optimized neural network were determined as 0.93232, 0.95854, and 0.98015, respectively. The results indicate that reasonable accuracy can be achieved by all three established models, while the genetic algorithm-optimized neural network achieves the highest accuracy among the three models.

**Figure 9:**
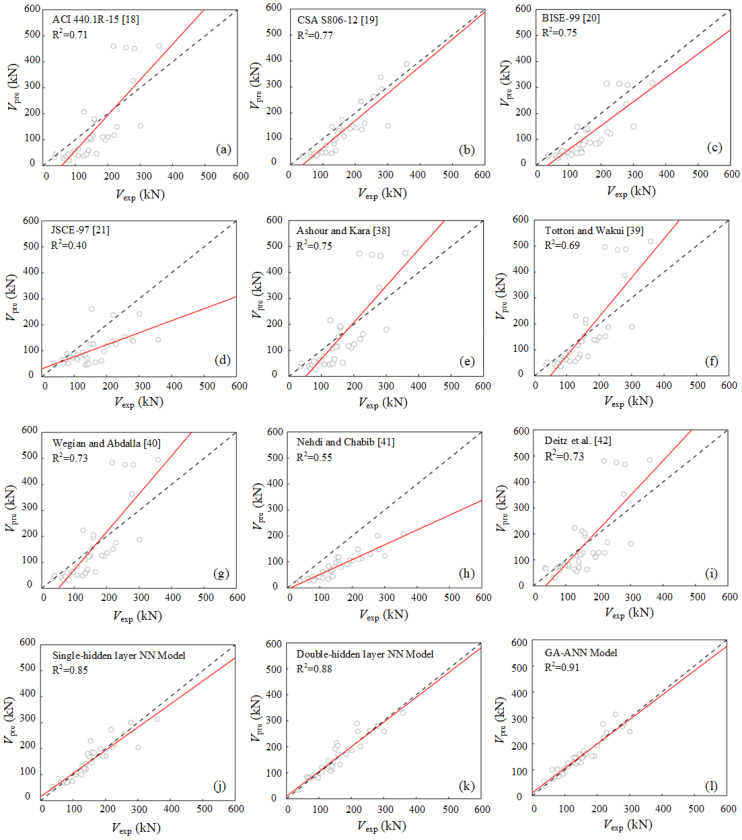
Comparison of shear capacity from the test and predicted results.

Moreover, in order to further evaluate the accuracy of the proposed models, the remaining 34 sets of test results were used to evaluate their generalization performance. The comparisons were made in terms of the shear capacity predicted by ACI 440.1R-2015 [18], CSA S806-12 [19], BISE 1999 [20], JSCE 1997 [21], and other existing models in the literature [38,39,40,41,42]. The scatter of the experimentally observed shear capacity, *V*_exp_, versus the predicted shear capacity, *V*_pre_, for the proposed model and other existing models are shown in Figure 9. Statistical parameters, namely, mean value (Mean), standard deviation (SD), coefficient of variation (COV), root-mean square error (RMSE), mean absolute error (MAE), and determination coefficients (R^2^), were used to evaluate the accuracy and efficiency of prediction. Table 3 summarizes these statistical parameters related to the ratio between the experimental and the predicted shear capacity, *V*_exp_/*V*_pre_.

As shown in Figure 9 and Table 3, the predictions of shear capacity of FRP-RC beams by existing design codes or guidelines tend to be conservative, with significant discreteness, and the statistical results in terms of Mean, SD, COV, RMSE, MAE, and R^2^ suggest the same observation. The predictive results of the proposed ML models are more consistent with the experimental results. Additionally, the newly developed GA-ANN model shows the highest prediction accuracy and lowest prediction errors in respect to all models for predicting shear capacity of FRP-RC beams, with a Mean of 0.99, R^2^ of 0.91, and RMSE of 22.60 kN. The most accurate code equation is that of CSA S806-12 [19], which has a Mean of 2.11, R^2^ of 0.77, and RMSE of 47.61 kN. However, the proposed GA-ANN model outperforms the CSA S806-12 [19] equation by improving the R^2^ value by 18.2% and the RMSE value by 52.5%. Thus, the proposed GA-ANN model can predict the shear capacity of FRP-RC beams with high accuracy and less error than available semi-empirical models over a wide range of design variables.

### 4.2. Coupling Effect of Parameters on Shear Capacity

According to the evaluation of the predictive results from three neural network models, the GA-ANN model was used to investigate the interaction among the design parameters and its effects on shear capacity of FRP-RC beams. In these parameters, the effective depth (d), shear span-to-depth ratio (a/d), concrete compressive strength ($f_{c}^{\prime }$), FRP longitudinal reinforcement ratio ($\rho_{fl}$), elastic modulus of FRP longitudinal reinforcement bars ($E_{fl}$), and FRP shear reinforcement ratio ($\rho_{fv}$) were determined to be the critical factors that govern the shear capacity of concrete beams reinforced with bars and stirrups, which is demonstrated in Figure 5. Therefore, the parameters were organized into three groups, i.e., geomatical configuration of beam (d and a/d), compressive strength of concrete and effective shear reinforcement ratio ($f_{c}^{\prime }$ and $\rho_{fv}$), and effective longitudinal reinforcement ratio ($\rho_{fl}$ and $E_{fl}$), which are presented in Table 4.

**Figure 10:**
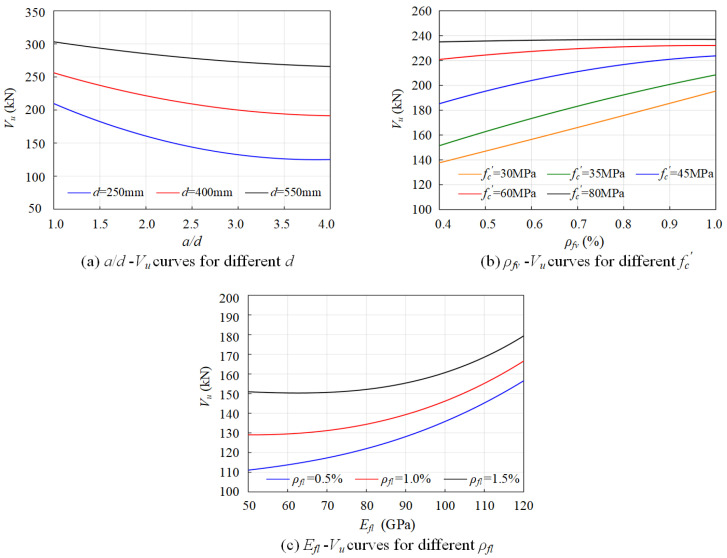
Coupling effects between the parameters on shear capacity of FRP-RC beams.

The effects of effective depth (d) and shear span-to-depth ratio (a/d) on shear capacity of concrete beams reinforced with FRP bars and stirrups are summarized in Figure 10a. With the increase in shear span-to-depth ratio (a/d), a reduction in shear capacity is observed for a concrete beam with various effective depths of beam. A linear correlation between the shear capacity and span-to-depth ratio is observed when the effective depth of beam is larger than 550 mm. At the same span-to-depth ratio, the reduction in shear capacity becomes more significant with decreased effective depth. For example, when the effective depth is taken as 550 mm, the shear capacity of the FRP-RC beam is decreased by 11.4% when the shear span-to-depth ratio varies from 1.0 to 4.0. However, the shear capacity is decreased by 40.7% when the effective depth is taken as 250 mm at the same shear span-to-depth ratio, suggesting a coupling effect between the shear span-to-depth ratio (a/d) and effective depth (d) on the shear capacity of the FRP-RC beam. Hence, the shear span-to-depth ratio becomes a critical parameter of the shear capacity of the FRP-RC beam when a low effective depth is adopted. This should be attributed to the arching effect in the concrete structure, in which the cracks develop significantly during the deformation of beam with mechanical interlock between the aggregates being reduced when the span-to-depth ratio increases. Such reduction is observed to be negligible when the shear span-to-depth ratio (*a*/*d*) reaches 3.5.

Figure 10b shows the effects of concrete compressive strength ($f_{c}^{\prime }$) and FRP shear reinforcement ratio ($\rho_{fv}$) on shear capacity of FRP-RC beams. It is noticed that at lower concrete grades, e.g., C30 and C35, the shear capacity of FRP-RC beams is increased linearly when higher $\rho_{fv}$ is adopted. With higher concrete grades, e.g., C45 and C60, the improvement of shear strength becomes nonlinear with increased shear reinforcement ratio. For example, when C30 concrete is adopted, the shear capacity of the FRP-RC beam is improved by 41.3% when the shear reinforcement ratio increases from 0.4% to 1.0%, while for C60 concrete, the shear capacity is only improved by 5.4% at the same range of shear reinforcement ratio. The finding suggests a reduced contribution of FRP stirrups with the increase in concrete compressive strength. Such observation is critical towards the shear design of an FRP-RC beam with the development of high-strength concrete (HPC), which could lead to the over-estimation of the contribution from FRP stirrups, while such a coupling effect between concrete grade and shear reinforcement ratio has not been considered in existing design guidelines.

The coupling effects between longitudinal reinforcement ratio ($\rho_{fl}$) and elastic modulus ($E_{fl}$) of FRP bars on shear capacity of FRP-RC beam are shown in Figure 10c. With the higher elastic modulus of longitudinal reinforcement, the shear capacity of the concrete beam is increased nonlinearly at various longitudinal reinforcement ratios. It is observed that at longitudinal reinforcement ratio, the enhancement of the elastic modulus of FRP bars becomes less effective in the improvement of shear capacity. For example, at the longitudinal reinforcement ratio of 0.5%, the shear capacity is increased by 39.3% when the $E_{fl}$ varies from 50 GPa to 120 GPa. However, the shear capacity is only increased by 17.8% at the longitudinal reinforcement ratio of 1.5% when the same range of $E_{fl}$ is adopted. This implies a reduced contribution of FRP longitudinal reinforcements with higher longitudinal reinforcement ratio.

Based on the above parametric study, it is observed that apart from the individual contribution of the determined parameters, there are coupling effects between the parameters on the shear capacity of FRP-RC beams, which should be considered in shear design of the corresponding structures. It should be noted that such coupling effects are usually neglected in the existing semi-empirical design formulas, as they are inherently determined based on the simplified mechanical model, while the coupling effects usually involve the interaction of the parameters with various rates of change and fluctuation for the entire scope of input parameters. The ANN models trained numerous preliminary linear and nonlinear models without any prior assumption regarding the shape and structure of the mathematical model, and it provides a powerful tool to address complexity and fluctuation nature of such issues as the prediction of shear capacity of FRP-RC beams for higher accuracy.

### 4.3. Data-Driven Regression Analysis

**Figure 11:**
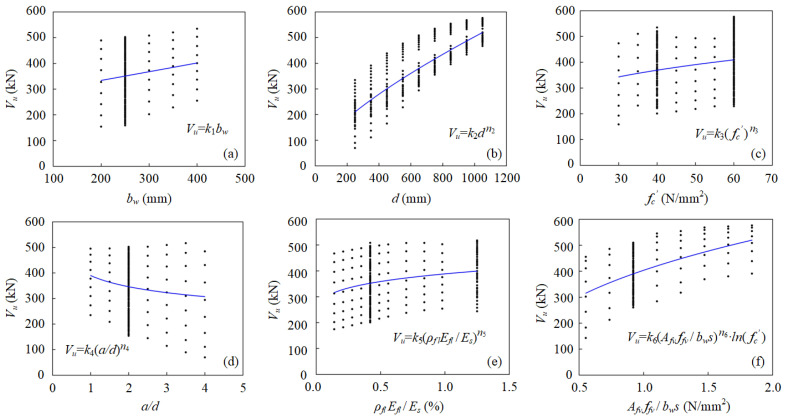
Determination of design parameters for the shear performance of FRP-RC beam.

Based on the GA-ANN model, a data-driven analysis was conducted for a precise predictive model of shear capacity of FRP-RC beams according to the critical design parameters. The validated GA-ANN model was utilized to generate a dataset of 400 results, considering the shear strength affected by the key parameters of the FRP-RC beam, i.e., beam width (b), effective depth (d), shear span-to-depth ratio (a/d), concrete compressive strength ($f_{c}^{\prime }$), FRP longitudinal reinforcement ratio ($\rho_{fl}$), FRP shear reinforcement ratio ($\rho_{fv}$), and elastic modulus of FRP longitudinal reinforcement bars ($E_{fl}$) and FRP stirrups ($E_{fv}$), whose value was selected according to the practical engineering design, as shown in Table 5. A predictive model was proposed according to Tottori and Wakui’s model [39] with further modification considering the coupling effects detected by GA-ANN model development, which is shown in Figure 11. The coupling effects from geomatical configuration of beam (d and a/d), compressive strength of concrete and effective shear reinforcement ratio ($f_{c}^{\prime }$ and $\rho_{fv}$), and effective longitudinal reinforcement ratio ($\rho_{fl}$ and $E_{fl}$) detected by the GA-ANN model have been addressed in the proposed predictive model, which is shown in the following equation.

(6) $$V=3\cdot b_{w}d^{0.7}\cdot {\left(f_{c}^{\prime }\frac{\rho_{fl}E_{fl}}{E_{s}}\right)}^{0.25}\cdot {\left(\frac{a}{d}\right)}^{-0.2}+{\left(\frac{A_{fv}f_{fv}}{b_{w}s}\right)}^{0.5}b_{w}d\cdot \left[ln{\left(f_{c}^{\prime }\right)}^{-1}+5\right]$$

**Figure 12:**
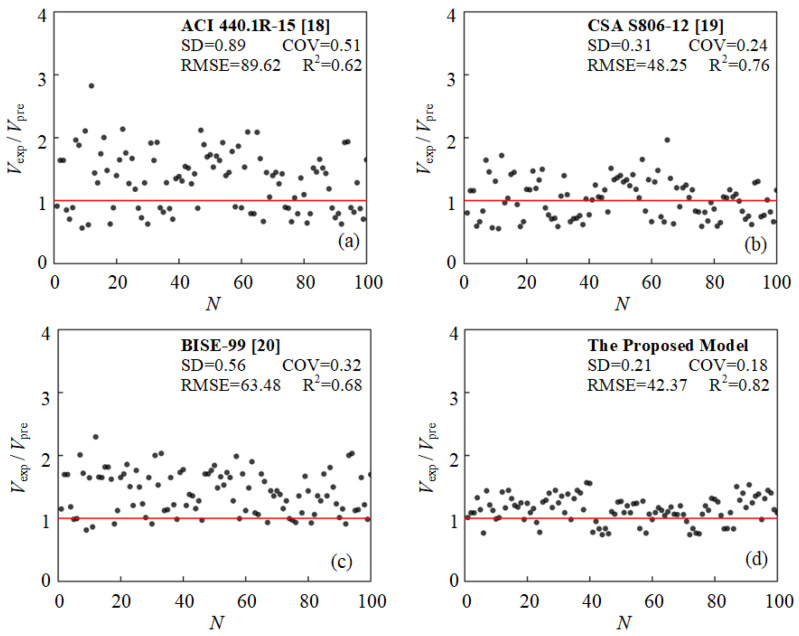
Comparison of prediction accuracy of proposed model and existing design codes.

In order to validate the proposed model and evaluate the accuracy of prediction, 100 experimental results were randomly selected from the dataset in Table 1, and the prediction accuracy was compared between the proposed model and the existing formula in the design guidelines, as shown in Figure 12. Similarly, the error analysis between the prediction of each model and experimental results is provided in Figure 12, including standard deviation (SD), covariance (COV), root-mean square error (RMSE), and determination coefficients (R^2^). According to the comparison, the most accurate prediction is achieved through the proposed model with the R^2^ and RMSE being determined as 0.82 and 42.37, respectively. The best code equation is that of CSA S806-12 [19], which has an R^2^ of 0.76 and RMSE of 48.25 kN. Thus, the proposed model outperforms CSA S806-12 [19] equation by improving the R^2^ value by 7.9% and the RMSE value by 12.1%. Moreover, neither over-estimation nor under-estimation is observed from prediction by the proposed model, implying that a reliable and practical estimation is achieved for the shear capacity of FRP-RC beams.

## 5. Conclusions

A machine learning method utilizing GA-ANN was developed from a database consisting of 120 test specimens to predict the shear capacity of concrete beams reinforced with FRP longitudinal reinforcements and stirrups. Through NID analysis, the critical factors affecting the shear capacity of concrete beams reinforced with FRP bars and stirrups were determined. The proposed GA-ANN model was validated by the existing experimental results as well as the design codes, and the coupling effects among the parameters were revealed. Furthermore, a simplified model extracted from GA-ANN was proposed and validated for the prediction of the shear capacity of FRP reinforced concrete beams. The developed model and corresponding analysis reported herein support the following conclusions:

(1) Existing design codes for the shear capacity of FRP-RC beams exhibit limited accuracy resulting from inconsistent expressions of different design parameters and overlooking the coupling effects between the geometrical configuration and mechanical properties of the reinforcements.
(2) Based on neural interpretation diagrams, the most critical design parameters that affect the shear strength of the FRP-RC beams are determined as beam width and depth, shear span-to-depth ratio, concrete compressive strength, longitudinal and shear reinforcement ratio, and elastic modulus of FRP reinforcements, which are in accordance with most of the existing design codes.
(3) The prediction accuracy of ANN and GA-ANN models in relation to the shear capacity of FRP-RC beams has been demonstrated through the comparison with the experimental results in the literature. The results of statistical measurements show that the proposed GA-ANN model outperforms the other equations in existing design codes and studies. The proposed GA-ANN model yields a Mean = 0.99, R^2^ = 0.91, and RMSE = 22.60 kN, which represents a 52.5% improvement in RMSE and 18.2% improvement in terms of R^2^ in respect to the CSA S806-12 equation as the best equation among the other design equations.
(4) According to the analysis of test and predictive results, the coupling effects between the geomatical configuration and mechanical properties of constitutive materials in FRP-RC have been observed. The shear strength of FRP-RC is increased linearly with the increase in the FRP stirrup reinforcement ratio when the compressive strength is lower than 45 MPa. With the higher concrete strength, the contribution of FRP stirrups to the shear resistance of FRP-RC beams becomes limited, leading to the overestimation of shear capacity of FRP-RC beams in existing design codes.
(5) Based on the GA-ANN model, a simplified design formula has been proposed, incorporating the coupling effects between the design parameters. The proposed model provides more reasonable predictive accuracy in terms of shear capacity of FRP-RC than that of existing design codes, according to the comparison with the experimental results.
(6) The proposed ANN and GA-ANN models are trained to predict the shear behavior of FRP-RC beams within the range of input variables considered. However, they may not demonstrate accuracy when extrapolating beyond this range. In this respect, more experiments need to be conducted to investigate the influences of design factors that affect shear behavior. Only when a sufficient number of data is considered will the proposed models be able to predict the shear capacity of FRP-RC beams in practical applications.

## Figures and Tables

**Table 1:** Shear design equations for FRP-RC beam with stirrups.

Reference	$\mathrm{Concrete}\;\mathrm{Shear}\;\mathrm{Contribution}\;V_{c}$	$\mathrm{Stirrup}\;\mathrm{Shear}\;\mathrm{Contribution}\;V_{s}$
ACI 440.1R-15 [18]	$V_{c}=\frac{2}{5}\sqrt{f_{c}^{\prime }}b_{w}\left(kd\right)$ $k=\sqrt{2\rho_{fl}n_{f}+{\left(\rho_{fl}n_{f}\right)}^{2}}-\rho_{fl}n_{f}$ $n_{f}=\frac{E_{fl}}{E_{c}}$	$V_{s}=\frac{A_{fv}f_{fv}d}{s}$ $f_{fv}=min\left\{0.004E_{fv},f_{fb},f_{fuv}\right\}$ $f_{fb}=\left[0.05\left(\frac{r_{b}}{d_{b}}\right)+0.3\right]f_{fuv}\leq f_{fuv}$
CSA S806-12 [19]	$V_{c}=k_{a}k_{s}\cdot 0.05\lambda k_{m}k_{r}{\left(f_{c}^{\prime }\right)}^{1/3}b_{w}d_{v}$ $k_{m}={\left(V_{\mu }d/M_{\mu }\right)}^{\frac{1}{2}}$ $k_{r}=1+{\left(E_{fl}\rho_{fl}\right)}^{\frac{1}{3}}$ $k_{a}=2.5/\left(M_{\mu }/V_{\mu }d\right)\geq$1 $k_{s}=750/\left(450+d\right)\leq 1$ $d_{v}=max\left\{0.9d,0.72h\right\}$	$V_{s}=\frac{A_{fv}f_{fv}d_{v}}{s}cot\theta$ $f_{fv}\leq 0.005E_{fv}$ $\theta =30{}^{\circ}+7000\varepsilon_{l}$ $\varepsilon_{l}=\frac{M_{\mu }/d_{v}+V_{\mu }+0.5N_{f}}{2E_{fv}A_{fv}}$
BISE-99 [20]	$V_{c}=0.79\sqrt[{4}]{\frac{400}{d}}\cdot \sqrt[{3}]{100\rho_{fl}E_{fl}/E_{s}}\cdot \sqrt[{3}]{\frac{f_{c}^{\prime }}{25}}b_{w}d$	$V_{s}=0.0025\frac{A_{fv}E_{fv}d}{s}$
JSCE-97 [21]	$V_{c}=\beta_{d}\beta_{p}\beta_{n}f_{vcd}b_{w}d$ $\beta_{d}={\left(\frac{1000}{d}\right)}^{\frac{1}{4}}\leq 1.5$ $\beta_{p}={\left(\frac{1000\rho_{fl}E_{fl}}{E_{s}}\right)}^{\frac{1}{3}}\leq 1.5$ $f_{vcd}=0.2\sqrt[{3}]{f_{c}^{\prime }}\leq 0.72\;MPa$	$V_{s}=\frac{A_{fv}E_{fv}\varepsilon_{fv}}{s}\cdot z$ $\varepsilon_{fv}=0.0001{\left(f_{mcd}^{\prime }\frac{\rho_{fl}E_{fl}}{\rho_{fv}E_{fv}}\right)}^{\frac{1}{2}}$ $f_{mcd}^{\prime }={\left(\frac{h}{0.3}\right)}^{-\frac{1}{10}}f_{c}^{\prime }$ z=d/1.15
Ashour and Kara [38]	$V_{c}=2.76{\left(f_{c}^{\prime }\cdot \frac{\rho_{fl}E_{fl}}{E_{s}}\cdot \frac{d}{a}\right)}^{1/3}b_{w}d$	$V_{s}=\frac{A_{fv}f_{fv}d}{s}$
Tottori and Wakui [39]	$V_{c}=0.2k_{1}k_{d}{\left(100f_{c}^{\prime }\frac{\rho_{fl}E_{fl}}{E_{s}}\right)}^{\frac{1}{3}}b_{w}d$ $k_{1}=\left(0.75+\frac{1.4d}{a}\right)$ $k_{d}={\left(\frac{d}{1000}\right)}^{\left(-\frac{1}{4}\right)}$	$V_{s}=\frac{A_{fv}f_{fv}d}{s}$
Wegian and Abdalla [40]	$V_{c}=2{\left(f_{c}^{\prime }\cdot \frac{\rho_{fl}E_{fl}}{E_{s}}\cdot \frac{d}{a}\right)}^{\frac{1}{3}}b_{w}d$	$V_{s}=\frac{A_{fv}f_{fv}d}{s}$
Nehdi and Chabib [41]	$V_{c}=2.1{\left(f_{c}^{\prime }\cdot \frac{\rho_{fl}E_{fl}}{E_{s}}\cdot \frac{d}{a}\right)}^{0.3}b_{w}d$	$V_{s}=0.5{\left(\rho_{fv}\cdot f_{fv}\right)}^{0.5}\cdot bd$
Deitz et al. [42]	$V_{c}=3\frac{E_{fl}}{E_{s}}\left(\sqrt{\frac{f_{c}^{\prime }}{6}}b_{w}d\right)$	$V_{s}=\frac{A_{fv}f_{fv}d}{s}$

Note: $f_{c}^{\prime }=$ compressive strength of concrete; $b_{w}$, h, and d= beam’s width, overall depth, and effective depth, respectively; a/d= shear span-to-depth ratio; $\rho_{fl}$ and $\rho_{fv}=$ FRP longitudinal reinforcement ratio and shear reinforcement ratio, respectively; $E_{c}$, $E_{s}$, $E_{fl}$, and $E_{fv}=$ elastic modulus of concrete, steel, FRP longitudinal reinforcement rebars, and FRP stirrups, respectively; $f_{ful}$ and $f_{fuv}=$ ultimate tensile strength of FRP longitudinal rebars and the straight portion of FRP stirrups, respectively; $f_{fv}=$ ultimate strength of FRP stirrups; $A_{fv}$ and s= the area and spacing of FRP stirrups, respectively; $r_{b}=$ the radius at the bent region of FRP stirrups; $d_{b}=$ the bar diameter of FRP stirrups; $V_{\mu }$ and $M_{\mu }=$ the ultimate shear and ultimate moment at a critical section, respectively; $N_{f}=$ the axial load ($N_{f}=0$ in this study).

**Table 2:** Details of experimental database.

Source	No.	Geometrical Characteristics				Concrete	Longitudinal Reinforcement				Shear Reinforcement				*V_exp_* (kN)
		*L* (mm)	*b* (mm)	*d* (mm)	*a/d*	*f_c_^′^* (MPa)	*Type_l_*	*ρ_fl_* (%)	*E_fl_* (GPa)	*f_ful_* (MPa)	*Type_v_*	*ρ_fv_* (%)	*E_fv_* (GPa)	*f_fuv_* (MPa)	
Nagasaka et al. [14]	22	600–1200	250	253	1.19–2.37	22.6–39.2	A	1.9	56	1295	A, C	0.5–1.48	44–112	481–903	158.9–359
Shehata et al. [15]	2	7000	135	470	3.19	50	C	1.25	137	2200	C, G	0.36, 1.07	41–137	640–1730	304.5–305
Tomlinson and Fam [16]	3	2900	150	245–270	4.07–4.5	51	B	0.39–0.51	70	1100	B	0.17	70	1100	36.4–53.5
Jumaa and Yousif [43]	1	1800	200	234	2.60	73.4	B	2.97	58	1200	B	0.63	56	1100	190.1
Razaqpur and Spadea [22]	6	2000	150	170	4.12	20–25.4	G	0.62–1.54	46–115	970–2000	G	0.29	46–115	970–2000	20.5–39.8
Tottori and Wakui [39]	3	1800	150–300	250–325	2.50–3.20	31.9–44.9	A, C	0.55–3.08	58–140	900–2100	A, C	0.04–0.13	53–144	500–1000	58–160
Maruyama and Zhao [44]	9	3000	150	250	3.00	30.5–38.3	C	0.5–2.11	94	1308	C	0.12–0.24	94	1308	59–119.5
Maruyama and Zhao [45]	4	1800–3750	150–450	250–750	2.50	29.5–34	C	1.04	100	1100	G	0.43–0.86	30	600	109.2–599.3
Zhao et al. [46]	5	1800–2000	150	250	2.00–4.00	34.3	C	1.51–3.03	105	1124	C, G	0.42	39	1100	73.8–126.6
Nakamura and Higai [47]	3	1500	200	250	3.00	34	G	1.61	29	751	G	0.18–0.35	31	828	61.9–100.8
Vijay et al. [48]	3	2500	150	265	1.89	31–44.8	G	0.67–1.43	54	655	G	0.56–0.83	142	655	116–127.8
Niewels [49]	7	2660–4500	300	404–441	3.02–3.71	29.1–48.3	G	3.25–3.98	44–63	480–1000	G	0.11–0.54	31–52	322–524	220–362
Ascione et al. [50]	6	2000	150	170	4.12	20–25.4	C, G	0.62–1.54	46–115	970–2000	C, G	0.28	46–115	970–2000	20.5–39.8
Chen et al. [51]	3	2100	200	310	1–1.94	30	C	0.97	175	2102.4	C	0.17–0.22	175	2102.4	197.7–215.4
Alsayed et al. [52]	5	1800	200	310	2.40–3.20	35	G	1.28–1.37	36–43	565	G	0.21–0.4	42	565	57.8–109
Said et al. [53]	9	1800	120	260	2.00	19.6–59.2	G	1.09–2.2	32	580	G	0.43–0.92	32	640	77–175.5
Li [54]	5	1000	150	215	1.16	28.3–49.3	B	0.96	55	1100	B	0.45–1.34	55	1100	135–184.4
Imjai et al. [55]	5	2300	150	220	3.50	50	G	1.22–1.3	45–60	700–1000	G	0.18–0.48	27	720	36.9–66.9
Hou [56]	1	2100	200	302	1.94	29.61	C	1	179	2196	C	0.17	159	914.5	150
Okamoto et al. [57]	11	600–1200	250	253	1.19–2.37	28.9–37.7	A	1.71	61	1167	A, C	0.51–1.5	61–113	822–903	158.9–359
Bentz et al. [58]	3	3050–7100	450	405–937	3.26	37.7	G	0.51–2.36	37	397	G	0.09	41	760	154–500
Duranovic et al. [59]	2	2300	150	210	2.44–3.65	40	G	1.36	45	1000	G	0.17	45	1000	49.8–67.4
Issa et al. [60]	2	3050	200	265	1.50–2.50	35.9	B	1.17–1.92	50	1060	B	0.65	53	1070	134.7–192.1
Mean	/	2056	202	279	2.59	35.26	/	1.59	66	1112.51	/	0.57	69	923	158.9
Standard deviation	/	1270.29	70.45	112.67	0.91	9.90	/	0.72	31.62	411.04	/	0.42	38.41	374.02	102.28
Minimum	/	600	120	170	1	19.6	/	0.39	29	397	/	0.04	27	322	20.5
Maximum	/	7100	450	937	4.5	73.4	/	3.98	179	2200	/	1.5	175	2102	599.3
Total	120	600–7100	120–450	170–937	1–4.5	19.6–73.4	/	0.39–3.98	29–179	397–2200	/	0.04–1.5	27–175	322–2102	20.5–599.3

Note: reinforcement type A, B, C, and G = aramid fiber-reinforced polymer (AFRP), basalt fiber-reinforced polymer (BFRP), carbon fiber-reinforced polymer (CFRP), and glass fiber-reinforced polymer (GFRP), respectively.

**Table 3:** Statistical parameters of *V*_exp_/*V*_pre_ for the proposed model and other existing model.

Model	Min	Max	Mean	SD (kN)	COV (%)	RMSE (kN)	MAE (kN)	R^2^
ACI 440.1R-15 [18]	0.47	4.05	1.77	0.910	51.48	90.46	73.95	0.71
CSA S806-12 [19]	0.83	2.11	1.31	0.299	22.88	47.61	31.04	0.77
BISE-99 [20]	0.69	2.83	1.69	0.573	34.41	66.37	56.37	0.75
JSCE-97 [21]	0.79	2.50	1.38	0.438	31.71	76.05	56.00	0.40
Ashour and Kara [38]	0.46	3.15	1.51	0.718	47.62	88.53	68.69	0.75
Tottori and Wakui [39]	0.44	2.32	1.28	0.523	40.84	91.89	65.85	0.69
Wegian and Abdalla [40]	0.45	2.64	1.40	0.619	44.27	89.64	67.27	0.73
Nehdi and Chabib [41]	0.94	2.58	1.51	0.378	25.06	56.61	47.16	0.55
Deitz et al. [42]	0.45	2.63	1.28	0.572	44.64	89.36	67.15	0.73
Single-hidden layer NN	0.67	1.47	1.02	0.179	17.57	29.26	21.14	0.85
Double-hidden layer NN	0.74	1.26	1.00	0.155	15.49	26.22	19.58	0.88
Genetic algorithm-optimized NN	0.66	1.26	0.99	0.145	14.81	22.60	16.14	0.91

Note: *V*_exp_ = experimentally observed shear capacity; *V*_pre_ = predicted shear capacity.

**Table 4:** Selected parameters and their ranges.

Group	b (mm)	d (mm)	a/d	$f_{c}^{\prime }$ (MPa)	$\rho_{fl}$ (%)	$E_{fl}$ (GPa)	$\rho_{fv}$ (%)	$E_{fv}$ (GPa)
1	250	250–550	1–4	30	2	56	0.5	112
2	250	250	2	30–60	2	56	0.4–1	46
3	250	250	2	40	0.5–1.5	50–120	0.5	46

**Table 5:** Value range of selected parameters in GA-ANN model.

b (mm)	d (mm)	a/d	$f_{c}^{\prime }$ (MPa)	$\rho_{fl}E_{fl}/E_{s}$ (%)	$\rho_{fv}$ (%)	$E_{fv}$ (GPa)
200–400	250–1050	1–4	30–60	0.14–0.98	0.2–1	40–120

## Data Availability

The data presented in this study are available upon request from the corresponding author.
